# Choices have consequences: the nexus between DNA repair pathways and genomic instability in cancer

**DOI:** 10.1186/s40169-016-0128-z

**Published:** 2016-12-05

**Authors:** Sonali Bhattacharjee, Saikat Nandi

**Affiliations:** Cold Spring Harbor Laboratory, Cold Spring Harbor, NY USA

**Keywords:** DNA damage, DNA repair, Genome editing, Genomic instability, Cancer, Chemotherapy, Double-strand break repair, Homologous recombination, Targeted therapy

## Abstract

**Background:**

The genome is under constant assault from a multitude of sources that can lead to the formation of DNA double-stand breaks (DSBs). DSBs are cytotoxic lesions, which if left unrepaired could lead to genomic instability, cancer and even cell death. However, erroneous repair of DSBs can lead to chromosomal rearrangements and loss of heterozygosity, which in turn can also cause cancer and cell death. Hence, although the repair of DSBs is crucial for the maintenance of genome integrity the process of repair need to be well regulated and closely monitored.

**Main body:**

The two most commonly used pathways to repair DSBs in higher eukaryotes include non-homologous end joining (NHEJ) and homologous recombination (HR). NHEJ is considered to be error-prone, intrinsically mutagenic quick fix remedy to seal together the broken DNA ends and restart replication. In contrast, HR is a high-fidelity process that has been very well conserved from phage to humans. Here we review HR and its sub-pathways. We discuss what factors determine the sub pathway choice including etiology of the DSB, chromatin structure at the break site, processing of the DSBs and the mechanisms regulating the sub-pathway choice. We also elaborate on the potential of targeting HR genes for cancer therapy and anticancer strategies.

**Conclusion:**

The DNA repair field is a vibrant one, and the stage is ripe for scrutinizing the potential treatment efficacy and future clinical applications of the pharmacological inhibitors of HR enzymes as mono- or combinatorial therapy regimes.

**Electronic supplementary material:**

The online version of this article (doi:10.1186/s40169-016-0128-z) contains supplementary material, which is available to authorized users.

## Background

DNA double strand breaks (DSBs) result as a consequence of the disassembly of the DNA double helix leading to the disruption of the stability of the genome. DSBs not only ensue from normal cellular metabolism, in the form of reactive oxygen species that can oxidize DNA bases [1, 2], but can also be generated during physiological processes like chromosome replication, meiotic recombination and DNA replication transcription collision [3–7]. Regardless of how DSBs are formed, faithful repair of these breaks are absolutely essential for maintenance of genome integrity. Failure to repair DSBs can lead to unwanted consequences, such as loss of genetic information, chromosomal rearrangements and even cell death. Cells have evolved with conserved recombination mediated genome editing pathways as a mean for repairing DSBs and restarting replication forks, thus allowing genome duplication to continue [8]. Recombination based mechanisms are crucial for both the repair and tolerance of DNA damage that vexes both strands of the double helix [9].

DNA double strand break repair (DSBR) pathways are generally classified based on whether sequence homology is used to join the broken DNA ends. Non-homologous end joining (NHEJ), which does not depend upon sequence homology, is the key repair pathway during the G0/G1 stages of the cell cycle [10]. During NHEJ, once a DSB is formed the broken ends are bound by Ku proteins (ku70 and ku80), which form a heterodimer and insulate the DNA ends from nucleolytic erosion [11, 12]. The Ku proteins foster direct ligation of the broken DNA ends by the specialized ligase complex Dnl4–Lif1 [12]. This complex can execute a blunt end ligation reaction on clean DNA ends, i.e. 3′-OH and 5′-phosphate groups. If the broken DNA ends are not clean, then further processing by nucleases and polymerases are necessary to ligate the loose ends [12]. However, in the midst of this process of genome editing, small deletions and insertions might be introduced at the junction site. This is why this pathway if often regarded to be an error-prone recovery mechanism [2, 13, 14].

In spite of the mutagenicity associated with NHEJ, its fast kinetics has a unique role in safeguarding genome integrity, particularly by suppressing chromosomal translocations [15]. A second NHEJ concomitant pathway often referred to as alternative-NHEJ (Alt-NHEJ), also known as Microhomology-mediated end joining (MMEJ), is another well-studied pathway for repairing DSBs in DNA [16]. The MMEJ repair pathway displays two diverging features from NHEJ; first is the use of 5–25 base pair (bp) microhomologous sequences during the alignment of the broken ends before religating them, and second is the slower kinetics of repair [15]. Much like NHEJ, MMEJ is frequently associated with chromosome anomalies such as deletions, translocations, inversions and other complex rearrangements. In contrast to NHEJ, there is an error-free DSBR pathway known as Homologous Recombination (HR) pathway where the cell employs a homologous DNA as template for the repair of the broken ends [17]. The homologous DNA may be a sister chromatid, a homologous chromosome or an ectopically located sequence. Further discussion on the detailed mechanisms of the repair systems mediated by NHEJ is beyond the scope of this review; instead we will focus on how DSBs are repaired error-free by HR, the various sub-types of HR and the molecular mechanisms regulating HR.

## Overview of homologous recombination

**Fig. 1:**
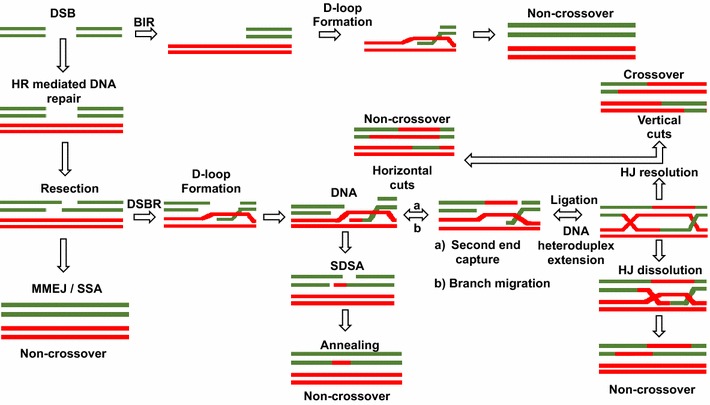
HR and its sub-pathways. This figure is adapted from [59, 83, 84]. Multiple HR pathways through which DSB’s can be repaired give rise to either crossovers or non-crossover recombinants. SSA and MMEJ are typically mutagenic as they result in the loss of intervening DNA sequences at the break site. In SDSA, Rad51-mediated strand invasion is terminated before second end capture and prevents the formation of dHJ. In DSBR, after the Rad51 nucleofilament invades homologous DNA molecule, new DNA synthesis is primed from the 3′ end of the invading strand resulting in second end capture and subsequent dHJ formation. dHJ can either by resolved by the action of junction resolving enzymes or alternatively by the dissolution of the structure. The recombinant outcome of each subpathway, crossover or non- crossover, is indicated

HR is the process by which DNA molecules of identical or nearly identical nucleotide sequences interact and exchange information that may or may not result in rearrangement of genetic information [18]. HR is widely considered as the major repair pathway in the mid-S and mid-G2 cell cycle phases, where DNA replication is maximum and therefore the sister template is accessible [18]. HR has three major steps: presynapsis, synapsis, and postsynapsis (Fig. 1). Mechanistically, in the presynaptic step, the DSB is resected enzymatically to generate ssDNA tails with 3′ ends [19, 20]. The ssDNA is then coated with Replication Protein A (RPA) to protect it from nuclease attacks as well as mediate the subsequent recombination steps [18]. With assistance from mediator proteins like Rad52, Rad55-57 and others, Rad51 displaces RPA and binds the ssDNA to form the nucleoprotein filament [18, 21, 22]. The Rad51 coated nucleoprotein filament then catalyzes strand invasion on a homologous region of another duplex to form an intermediate structure referred to as the displacement loop (D-loop), following which the invading 3′ end primes DNA synthesis [18]. The formation of the junction intermediate is the synapsis stage. During post-synapsis, junction intermediates are processed to form mature recombinant products [23]. How the intermediates are processed largely depends on the nature of the break and the genomic environment [23]. There are six major pathways using HR, also referred to as HR sub-pathways [18]. The models for the general scheme of HR and its sub-pathways derive mainly from analyses of patterns of segregation of genetic markers in fungal crosses [24, 25]. These sub-pathways include (1) The canonical DSBR pathway, (2) synthesis-dependent strand annealing (SDSA), (3) break-induced replication (BIR), (4) single-strand annealing (SSA), (5) alternative (microhomology-mediated) end joining (alt-EJ/MMEJ) and (6) RNA-templated DSBR.

## Major DNA repair pathways using homologous recombination

### Double strand break repair (DSBR) pathway

One of the most preeminent models of HR is the DSBR model published by Szostak et al. [26]. This model proposes that once a DSB is formed, recombination is initiated at the site of the break [27]. This triggers the resection of DSB by a nuclease, degrading the 5′ ended strand to form a DNA molecule with a ssDNA tail terminating with a 3′OH moiety [18]. The ssDNA coated with RPA is then replaced by the Rad51 recombinase in a reaction facilitated by mediator proteins Rad52, Rad55–57 and others [28]. The Rad51 protein coated on the ssDNA forming a nucleoprotein filament then locates and pairs with an intact homologous DNA molecule, followed by which the ssDNA tail invades the duplex to form a structure called the D-loop with the assistance of the DNA translocases Rad54 [29]. DNA synthesis is primed from 3′ end of the invading strand resulting in the extension of the D-loop, which exposes DNA that is complementary to the ssDNA tail at the other end of the break. This tail anneals to the D-loop in a process that is referred to as second end capture (SCE) [29]. Further DNA synthesis and the sealing of strand nicks by DNA ligases allows linkage of the two DNA molecules by a two four-way DNA junction. These junctions are called Holliday junctions (HJ) [30]. To facilitate segregation of the two HJs, also referred to as the double Holliday Junction (dHJ), the four-way DNA structure undergoes endonucleatic cleavage of a pair of symmetrically related strand at each junction. This fractures the connection between the two DNA molecules resulting in the formation of nicked linear duplexes and can be repaired by DNA ligases [30]. A notable hallmark of DSBR is that the choice of strand cleaved at each junction determines the type of recombinant DNA molecule generated (Fig. 1). If different pair of strands are cleaved at each junction, there is a reciprocal exchange of DNA arm flanking the site of recombination generating crossover recombinants (CO). However, if the same strands are cleaved at each junction the DNA arms do not exchange and this type of product is called a non-crossover (NCO) [23, 31].

### Synthesis-dependent strand annealing (SDSA) pathway

Like the DSBR model, recombination by SDSA is initiated at DSBs, and the 3′-end of the invading strand functions as a primer for DNA synthesis (Fig. 1). However, instead of dHJ formation, in the SDSA pathway a short D-loop is formed, that is dissolved before the SCE occurs. The newly synthesized strand is then annealed to the complementary end of the second tail. As a consequence, no dHJ structure is formed leading to exclusively NCO recombinant products [32]. In yeast mitotic cells there are two helicases that promotes SDSA, and in doing so suppresses CO recombination [32]. Both these helicases, Srs2 and Mph1 bind Rad51 and disrupt the Rad51 presynaptic filaments thereby dissociating the D-loop [32–36].

### Break-induced replication (BIR) pathway

Break-induced replication is a preferred pathway of repairing DSBs that are one-sided and hence only one of the end shares homology with an intact DNA sequence (Fig. 1) [37]. Albeit mutagenic, BIR is useful for restarting replication at broken forks. In the absence of SCE, extension of the invading strand accompanied by branch migration of the D-loop continues until the end of the template chromosome or termination by meeting the converging fork [38].

### Single-strand annealing (SSA) pathway

Single-strand annealing is also a mutagenic HR sub-pathway. The most common substrate for the SSA pathway is DSBs that are flanked by homologous sequences such as found in regions of repeated DNA sequences (Fig. 1). It is a unique HR sub-pathway in that it does not require the Rad51-dependent strand invasion step for homologous strand pairing. Instead, it is mediated by an additional Rad52 enzymatic activity that is unique for ssDNA annealing [29, 39, 40]. Resection of the DSB exposes complementary 3′ ssDNA tails that anneal to each other. The non-homologous DNA between the annealed repeats is flipped out, forming 3′ flaps, which is then cleaved by the Rad1–Rad10 complex [40]. This results in the deletion of one of the repeats as well as the intervening region [40, 41]. Since, repetitive elements are numerous in the human genome; consequently SSA could mediate large-scale rearrangements that cause deletions of sequences located between the repeats.

### Alternative (microhomology-mediated) end joining (alt-EJ/MMEJ)

Microhomology-mediated end joining is also a mutagenic repair pathway. Although related to NHEJ, this pathway shares key mechanistic attributes with HR [42]. MMEJ, like HR, operates predominantly during S-phase of the cell cycle, in a Ku- and DNA-PK-independent fashion. MMEJ begins with the resection of 5′ DNA ends at the DSB, leaving behind 3′ ssDNA [42]. Although MMEJ and DSBR employ the same end processing enzymes, subsequent steps diverge. MMEJ occurs when end-resection exposes micro-homologies of 10–25 bp that enables ssDNA to anneal [42]. This creates a substrate that, following removal of non-annealed DNA ends, is proficient for gap filling and ligation, like in SSA repair (Fig. 1) [43, 44].

### RNA-templated DSBR

A crucial limitation of HR is that it requires an undamaged template, to restore the information on the damaged duplex. If the DSB arises at a locus that has been replicated, a template is available in the form of a sister chromatid. Thus, while HR allows for accurate repair by a sister chromatid in mitotic cells, it is limited to the S and G2 phases of the cell cycle. If, however, the DSB occurs before replication, no such DNA template is available. The RNA transcript can at least in principle act as a repair template, this is referred to as RNA-templated DNA repair [45]. A role for RNA in DNA repair via NHEJ following reverse transcription into complementary DNA (cDNA) has been illustrated [46]. In this section, we will discuss the current findings in DSBR by RNA via HR mechanism.

### RNA-templated DSBR mediated by HR

**Fig. 2:**
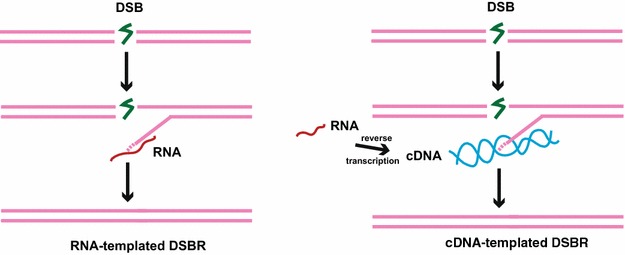
Models explaining RNA-templated DSBR and cDNA-templated DSBR. This figure is adapted from [85]

Since RNA molecules are complementary copies of the DNA from which they are derived, several laboratories have tried to elucidate a direct role of RNA as a template in DNA repair (Fig. 2). More recently, studies in both yeast and human cells have shown evidence of direct RNA-templated DNA repair by HR. In the yeast *Saccharomyces cerevisiae,* Resnick et al. measured the repair of a linearized *leu2* marker locus in yeast chromosomal DNA leading to Leu+ transformants through recombination with RNA oligonucleotides complementary to the broken ends that were transformed into the cells [45]. This combined with the observations that in vitro, the yeast replicative DNA polymerases such as α and δ can replicate short RNA template tracts led them to conclude that RNA oligonucleotides can directly template the repair of a DSB in yeast cells [45]. Subsequently, Storici et al. showed that direct RNA-templated DNA repair by HR is not restricted to yeast but is an evolutionarily conserved phenomenon. They demonstrated the repair of a chromosomal break generated within a copy of the GFP gene cut by the I-SceI endonuclease and then repaired by RNA-containing and RNA-only oligonucleotides in the genome of human HEK-293 cells [47]. These findings demonstrate that the genetic information on transcript RNA can be used to repair DSBs (Fig. 2). RNA-dependent DSBR much like DNA-dependent DSBR works by the process of HR and involves the RAD52 epistasis group of genes, encoding proteins such as Rad51, Rad52 and XRCC3 [48].

### HR mediated by cDNA intermediates

cDNA is a short dsDNA intermediate, which results from reverse transcription of endogenous cellular RNA. Since like the precursor RNA, cDNA may have sequence homology with regions around a DSB, it raises the possibility that a cDNA reverse transcript might mediate DSBR (Fig. 2). This was demonstrated by a system developed by Derr et al., the plasmid used in the study carries a *his3* reporter gene whose coding sequence is interrupted by an artificial intron inserted at a unique *MscI* site in the antisense orientation relative to the *HIS3* promoter [49]. The gene cassette is under the control of an inducible galactose promoter, which upon induction produces a sliceable *his3* transcript [49]. This transcript can be subsequently reverse transcribed by Ty and integrated into the genome resulting in *His*+ prototrophic cells in a strain containing chromosomal *his3* deletion [49]. Derr et al. observed *His*+ prototrophic cells at a frequency of around 200 per 109, which could only result from recombination between the *HIS3* cDNA and chromosomal *his3*-∆*Msc1 allele* [49]. These findings demonstrate that like with the RNA transcript, genetic information on cDNA molecule can be used to repair DSBs (Fig. 2).

### The regulation of Homologous Recombination

Although HR is an important DNA repair mechanism that protects genome integrity, it also has the ability to threaten the genome and cause cancer or cell death. As described above a key feature of HR is that it can use any homologous DNA sequences in the genome as substrate. Use of the homologous chromosome instead of the sister chromatid in diploid organisms can potentially lead to loss of heterozygosity [50]. Faulty recombination can result in translocation events as well as expansion and contraction of tandem arrays demonstrating the need for the tight regulation of recombination [27]. Interestingly, it is precisely the ability to promote genome rearrangements that is used in some situations to beneficial effect, raising the question as to how cells have evolved the mandatory controls to grant beneficial rearrangements but at the same time inhibiting those that would be harmful [51]. Since HR is limited to S and G2 phases, its regulation is linked to the cell cycle apparatus. DSB formation during late S-phase or G2 phase initiates a DNA damage checkpoint that forestalls cells from progressing through mitosis until DNA is repaired [52]. Additionally, as stated earlier, whether DSBs are ‘clean’ or ‘dirty’ can influence both the pathway used to repair a DSB, and the kinetics of repair. The commitment to HR is established after the CDK1-dependent DSB resection [53, 54]. CDK1-dependent phosphorylation of CtIP is crucial for its recruitment to the break site where it activates the Mre11 nuclease [55, 56]. In the absence of phosphorylated CtIP, NHEJ is the preferred pathway for DSBR because the unresected DSBs act as perfect substrates for binding of Ku proteins that initiate NHEJ [53].

Another key focus of the regulation of HR is the formation of the presynaptic filament and the resolution of junction intermediates. Once the DNA ends are resected and ssDNA is formed, it is rapidly bound by RPA, which is important to protect the substrate from exonucleases and the formation of secondary DNA structures, but blocks its access from Rad51 [56]. Important positive mediators of nucleofilament formation include, Rad52, other Rad51 paralogs and BRCA2 in mammalian cells that have roles in promoting presynaptic filament formation by helping overcome the inhibitory effect of RPA [21, 57]. The mediators act by stabilizing the ATPase activity of Rad51 and in turn enhancing its ability to bind ssDNA [58, 59]. Conversely, many factors negatively regulate RAD51 nucleofilament formation to ensure strand exchange regulation. For instance, once RAD51 is bound, it is actively debarked by helicase Fbh1 [56]. In *S. cerevisiae*, Srs2 negatively regulates HR by removing Rad51 from presynaptic filaments through an ATP-hydrolyzing activity coupled to DNA unwinding and channeling lesions into the non-recombinogenic Rad6–Rad18 mediated post replication repair pathway [33, 60, 61].

The checkpoint protein ATM regulates the synaptic step by controlling the activation of Rad54 [62, 63]. Rad54 activation is crucial for Rad51 mediated homology search, strand invasion, D-loop formation and junction resolution [62].

Another primary focus of HR regulation is the fate of HJ intermediates. In mitotic cells, COs are strongly suppressed by tightly modulating the orientation of dHJ cleavage. Several DNA helicases have been implicated in regulating HR by “resolving” recombination intermediates as NCOs. RecQ helicases in both yeast and humans have been shown to negatively regulate CO recombinants by modulating various steps of HR like displacing RAD51 from ssDNA in an ATP-dependent manner, displacing D-loops before SCE and even dissolution of the dHJ [64–66]. Members of the FANCM family of helicases have been reported to promote SDSA-mediated NCO product formation by disrupting RAD51 coated D-loops [32, 67–69].

### Homologous recombination in cancer diagnosis and treatment

As discussed earlier, HR and its sub pathways play a key role in protecting the genome against instability. In doing so HR plays a crucial role in cancer cell formation and its therapeutic response. Due to its role in maintaining genome integrity, HR is often considered the dernier resort in preventing tumorigenesis [70]. On the other hand, unregulated HR or erroneous repair or replicative bypass of lesions can also lead to chromosomal translocations and genomic rearrangements, which results in the dominant negative effect of HR [71]. Many mutations in HR genes have been associated with cancers, e.g., BRCA1 and BRCA2 in breast and ovarian cancer [72]; RAD54 in colon cancer [73]; MRN complex in melanoma, ovarian, colorectal, and head and neck cancer [74]; RECQL4 in skin carcinomas [75] and other well-characterized helicases. This evidence makes it abundantly clear that HR defects and defects in DNA damage response (DDR) cause cancer and are common in cancer cells. Taken together, this suggests that targeting HR is a valid anticancer strategy.

### Combination therapy: targeting synthetic lethal interactions

Promising data from both preclinical and clinical studies have given rise to the powerful concept for therapeutic combinations of DNA repair inhibitors with DNA damaging anticancer agent [76]. This is known as conditional (synthetic) lethality or combination therapy. This strategy is rooted in the principles from yeast genetics where mutants of either two related genes are viable, but loss of both genes functioning in a redundant pathway causes cell death [76]. This strategy allows for the targeted killing of tumor cells by inhibiting genes, which are synthetic lethal, with a mutated tumor suppressor gene by developing cocktails of cytotoxic agents and inhibitors of DNA repair [77]. The best-characterized candidates in this principle are the PARP inhibitors, which are selectively active in BRCA2-deficient tumors. Since BRCA2-deficient tumors cannot repair DSBs by HR, they rely on NHEJ. PARP is a central component in the base excision repair (BER) pathway and PARP inhibition abolishes the BER pathway, leading to a further accumulation of unresolved SSBs that convert to DSBs during S phase. In these tumor cells, although NHEJ is active, it cannot compensate for the loss of both HR and BER. This leads to the accumulation of recombinogenic lesions and other errors that cause the collapse of replication forks and lead to cell death [78, 79]. Another example of rationale drug combination is the association of checkpoint protein and DNA-dependent protein kinase (DNA-PK) inhibitors. DNA-PK, belongs to the PI3-K related kinase family and is a component of the NHEJ pathway [80, 81]. DNA-PK inhibitors have been shown to have a synthetic lethal relationship with ATM-deficient tumors [80]. Several inhibitors identified using the combinatorial therapy approach are now in various stages of clinical trials. For instance, Helleday laboratory has reported promising data using PARP inhibitors that can selectively kill BRCA1 and BRCA2 defective tumors [82]. FDA has recently accepted a drug named Rucaparib for priority review for the treatment of advanced mutant BRCA ovarian cancer (S1). A PARP inhibitor drug Lynparza (Olaparib) has also been recently approved by the FDA for the treatment of patients with germline BRCA-mutated advanced ovarian cancer (S2). Interestingly, myChoice HRD test developed by Myriad Genetics is a homologous recombination deficiency biomarker that indicates the inability of cancer cells to repair dsDNA breaks, resulting in increased susceptibility to PARP inhibitors and platinum-based therapies (S3). Overall, the link between cancer and HR provides the basis for the use of inhibitors against HR genes in therapy (Additional file 1).

## Future perspectives

The implication of understanding the molecular details of HR in determining vital targets for cancer therapies is the perfect marriage of basic research and translational science. The HR pathway is an appealing target for the development of inhibitors because cancer cells rely on HR proteins for repair of DSBs, which arise as a consequence of chemotherapy. The strategy of synthetic lethality has been validated by research and clinical trials. The recent success of PARP inhibitors in BRCA2-deficient breast and ovarian cancer has made the promise of inhibitors of DNA repair to transform the therapeutic landscape in cancer more apparent. Better characterization of DNA repair proteins will allow therapies that specifically target selected repair pathways for more effective repair inhibitors and to achieve cancer cure. Challenges in this field lie in the heterogeneity in the levels of HR proteins in different types of tumors, the lack of reliable biomarkers to validate resistance to the inhibitors, development of early molecular diagnostic tools and the evolution of resistance by cancer cells from acquiring additional mutations. Although more research is required to develop more targeted inhibitors of HR, there is no denying that further studies on the mechanistic roles of enzymes mediating DNA repair pathways have immense therapeutic potential.

## Additional file

**Additional file 1.** Supplementary references.

## References

[1] Hori A (2009). Reactive oxygen species regulate DNA copy number in isolated yeast mitochondria by triggering recombination-mediated replication. Nucleic Acids Res.

[2] Symington LS, Gautier J (2011). Double-strand break end resection and repair pathway choice. Annu Rev Genet.

[3] Aguilera A, Gaillard H (2014). Transcription and recombination: when RNA meets DNA. Cold Spring Harb Perspect Biol.

[4] Mehta A, Haber JE (2014). Sources of DNA double-strand breaks and models of recombinational DNA repair. Cold Spring Harb Perspect Biol.

[5] Syeda AH, Hawkins M, McGlynn P (2014). Recombination and replication. Cold Spring Harb Perspect Biol.

[6] Zaratiegui M (2011). RNAi promotes heterochromatic silencing through replication-coupled release of RNA Pol II. Nature.

[7] Castel SE (2014). Dicer promotes transcription termination at sites of replication stress to maintain genome stability. Cell.

[8] Branzei D, Szakal B (2016). DNA damage tolerance by recombination: molecular pathways and DNA structures. DNA Repair (Amst).

[9] Cahill D, Connor B, Carney JP (2006). Mechanisms of eukaryotic DNA double strand break repair. Front Biosci.

[10] Chiruvella KK, Liang Z, Wilson TE (2013). Repair of double-strand breaks by end joining. Cold Spring Harb Perspect Biol.

[11] Kass EM, Jasin M (2010). Collaboration and competition between DNA double-strand break repair pathways. FEBS Lett.

[12] Ma Y (2005). Repair of double-strand DNA breaks by the human nonhomologous DNA end joining pathway: the iterative processing model. Cell Cycle.

[13] Daley JM (2005). Nonhomologous end joining in yeast. Annu Rev Genet.

[14] Aylon Y, Kupiec M (2004). DSB repair: the yeast paradigm. DNA Repair (Amst).

[15] Difilippantonio MJ (2000). DNA repair protein Ku80 suppresses chromosomal aberrations and malignant transformation. Nature.

[16] Decottignies A (2013). Alternative end-joining mechanisms: a historical perspective. Front Genet.

[17] Hefferin ML, Tomkinson AE (2005). Mechanism of DNA double-strand break repair by non-homologous end joining. DNA Repair (Amst).

[18] San Filippo J, Sung P, Klein H (2008). Mechanism of eukaryotic homologous recombination. Annu Rev Biochem.

[19] Paull TT, Gellert M (1998). The 3′ to 5′ exonuclease activity of Mre 11 facilitates repair of DNA double-strand breaks. Mol Cell.

[20] Paull TT, Gellert M (1999). Nbs1 potentiates ATP-driven DNA unwinding and endonuclease cleavage by the Mre11/Rad50 complex. Genes Dev.

[21] Sugiyama T, Kowalczykowski SC (2002). Rad52 protein associates with replication protein A (RPA)-single-stranded DNA to accelerate Rad51-mediated displacement of RPA and presynaptic complex formation. J Biol Chem.

[22] Tsukamoto Y, Kato J, Ikeda H (1996). Effects of mutations of RAD50, RAD51, RAD52, and related genes on illegitimate recombination in *Saccharomyces cerevisiae*. Genetics.

[23] Hunter N, Kleckner N (2001). The single-end invasion: an asymmetric intermediate at the double-strand break to double-holliday junction transition of meiotic recombination. Cell.

[24] Hastings PJ (1992). Mechanism and control of recombination in fungi. Mutat Res.

[25] Orr-Weaver TL, Szostak JW (1985). Fungal recombination. Microbiol Rev.

[26] Szostak JW (1983). The double-strand-break repair model for recombination. Cell.

[27] Sung P, Klein H (2006). Mechanism of homologous recombination: mediators and helicases take on regulatory functions. Nat Rev Mol Cell Biol.

[28] Sung P (1997). Function of yeast Rad52 protein as a mediator between replication protein A and the Rad51 recombinase. J Biol Chem.

[29] Symington LS, Rothstein R, Lisby M (2014). Mechanisms and regulation of mitotic recombination in *Saccharomyces cerevisiae*. Genetics.

[30] Raji H, Hartsuiker E (2006). Double-strand break repair and homologous recombination in *Schizosaccharomyces pombe*. Yeast.

[31] Lorenz A (2012). The fission yeast FANCM ortholog directs non-crossover recombination during meiosis. Science.

[32] Sun W (2008). The FANCM ortholog Fml1 promotes recombination at stalled replication forks and limits crossing over during DNA double-strand break repair. Mol Cell.

[33] Krejci L (2003). DNA helicase Srs2 disrupts the Rad51 presynaptic filament. Nature.

[34] Veaute X (2003). The Srs2 helicase prevents recombination by disrupting Rad51 nucleoprotein filaments. Nature.

[35] Prakash R (2009). Yeast Mph1 helicase dissociates Rad51-made D-loops: implications for crossover control in mitotic recombination. Genes Dev.

[36] Bhattacharjee S (2013). MHF1-2/CENP-S-X performs distinct roles in centromere metabolism and genetic recombination. Open Biol.

[37] Jain S (2009). A recombination execution checkpoint regulates the choice of homologous recombination pathway during DNA double-strand break repair. Genes Dev.

[38] Michel B (2000). Replication fork arrest and DNA recombination. Trends Biochem Sci.

[39] Lisby M, Rothstein R (2009). Choreography of recombination proteins during the DNA damage response. DNA Repair (Amst).

[40] Paques F, Haber JE (1999). Multiple pathways of recombination induced by double-strand breaks in *Saccharomyces cerevisiae*. Microbiol Mol Biol Rev.

[41] Pfeiffer P (2004). Pathways of DNA double-strand break repair and their impact on the prevention and formation of chromosomal aberrations. Cytogenet Genome Res.

[42] Truong LN (2013). Microhomology-mediated end joining and homologous recombination share the initial end resection step to repair DNA double-strand breaks in mammalian cells. Proc Natl Acad Sci USA.

[43] Frankenberg-Schwager M (2009). Single-strand annealing, conservative homologous recombination, nonhomologous DNA end joining, and the cell cycle-dependent repair of DNA double-strand breaks induced by sparsely or densely ionizing radiation. Radiat Res.

[44] Sfeir A, Symington LS (2015). Microhomology-mediated end joining: a back-up survival mechanism or dedicated pathway?. Trends Biochem Sci.

[45] Storici F (2007). RNA-templated DNA repair. Nature.

[46] Moore JK, Haber JE (1996). Capture of retrotransposon DNA at the sites of chromosomal double-strand breaks. Nature.

[47] Shen Y (2011). RNA-driven genetic changes in bacteria and in human cells. Mutat Res.

[48] Wahba L, Gore SK, Koshland D (2013). The homologous recombination machinery modulates the formation of RNA-DNA hybrids and associated chromosome instability. Elife.

[49] Derr LK, Strathern JN (1993). A role for reverse transcripts in gene conversion. Nature.

[50] Colavito S, Prakash R, Sung P (2010). Promotion and regulation of homologous recombination by DNA helicases. Methods.

[51] Shrivastav M, De Haro LP, Nickoloff JA (2008). Regulation of DNA double-strand break repair pathway choice. Cell Res.

[52] Tichy ED (2010). Mouse embryonic stem cells, but not somatic cells, predominantly use homologous recombination to repair double-strand DNA breaks. Stem Cells Dev.

[53] Peterson SE (2013). Activation of DSB processing requires phosphorylation of CtIP by ATR. Mol Cell.

[54] Peterson SE (2011). Cdk1 uncouples CtIP-dependent resection and Rad51 filament formation during M-phase double-strand break repair. J Cell Biol.

[55] Huertas P (2008). CDK targets Sae2 to control DNA-end resection and homologous recombination. Nature.

[56] Osman F (2005). The F-Box DNA helicase Fbh1 prevents Rhp51-dependent recombination without mediator proteins. Mol Cell Biol.

[57] Esashi F (2007). Stabilization of RAD51 nucleoprotein filaments by the C-terminal region of BRCA2. Nat Struct Mol Biol.

[58] Ayoub N (2009). The carboxyl terminus of Brca2 links the disassembly of Rad51 complexes to mitotic entry. Curr Biol.

[59] Li X, Heyer WD (2008). Homologous recombination in DNA repair and DNA damage tolerance. Cell Res.

[60] Aboussekhra A (1989). RADH, a gene of *Saccharomyces cerevisiae* encoding a putative DNA helicase involved in DNA repair. Characteristics of radH mutants and sequence of the gene. Nucleic Acids Res.

[61] Rong L (1991). The hyper-gene conversion hpr5-1 mutation of *Saccharomyces cerevisiae* is an allele of the SRS2/RADH gene. Genetics.

[62] Bakr A (2015). Involvement of ATM in homologous recombination after end resection and RAD51 nucleofilament formation. Nucleic Acids Res.

[63] Lisby M, Rothstein R (2004). DNA damage checkpoint and repair centers. Curr Opin Cell Biol.

[64] Gravel S (2008). DNA helicases Sgs1 and BLM promote DNA double-strand break resection. Genes Dev.

[65] Grabarz A (2013). A role for BLM in double-strand break repair pathway choice: prevention of CtIP/Mre11-mediated alternative nonhomologous end-joining. Cell Rep.

[66] Paliwal S (2014). Human RECQ5 helicase promotes repair of DNA double-strand breaks by synthesis-dependent strand annealing. Nucleic Acids Res.

[67] Gari K (2008). Remodeling of DNA replication structures by the branch point translocase FANCM. Proc Natl Acad Sci USA.

[68] Stafa A (2014). Template switching during break-induced replication is promoted by the Mph1 helicase in *Saccharomyces cerevisiae*. Genetics.

[69] Nandi S, Whitby MC (2012). The ATPase activity of Fml1 is essential for its roles in homologous recombination and DNA repair. Nucleic Acids Res.

[70] Patel KJ (1998). Involvement of Brca2 in DNA repair. Mol Cell.

[71] Mohindra A (2002). Defects in homologous recombination repair in mismatch-repair-deficient tumour cell lines. Hum Mol Genet.

[72] Wooster R (1995). Identification of the breast cancer susceptibility gene BRCA2. Nature.

[73] Hiramoto T (1999). Mutations of a novel human RAD54 homologue, RAD54B, in primary cancer. Oncogene.

[74] Mosor M (2006). Association of the heterozygous germline I171V mutation of the NBS1 gene with childhood acute lymphoblastic leukemia. Leukemia.

[75] Mohaghegh P, Hickson ID (2001). DNA helicase deficiencies associated with cancer predisposition and premature ageing disorders. Hum Mol Genet.

[76] Helleday T (2008). DNA repair pathways as targets for cancer therapy. Nat Rev Cancer.

[77] Rehman FL, Lord CJ, Ashworth A (2010). Synthetic lethal approaches to breast cancer therapy. Nat Rev Clin Oncol.

[78] Ratnam K, Low JA (2007). Current development of clinical inhibitors of poly(ADP-ribose) polymerase in oncology. Clin Cancer Res.

[79] Zaremba T, Curtin NJ (2007). PARP inhibitor development for systemic cancer targeting. Anticancer Agents Med Chem.

[80] Salles B (2006). The DNA repair complex DNA-PK, a pharmacological target in cancer chemotherapy and radiotherapy. Pathol Biol (Paris).

[81] Gurley KE, Kemp CJ (2001). Synthetic lethality between mutation in Atm and DNA-PK(cs) during murine embryogenesis. Curr Biol.

[82] Bryant HE (2005). Specific killing of BRCA2-deficient tumours with inhibitors of poly(ADP-ribose) polymerase. Nature.

[83] Wu L, Hickson ID (2006). DNA helicases required for homologous recombination and repair of damaged replication forks. Annu Rev Genet.

[84] Cejka P (2010). DNA end resection by Dna2-Sgs1-RPA and its stimulation by Top3-Rmi1 and Mre11-Rad50-Xrs2. Nature.

[85] Meers C, Keskin H, Storici F (2016). DNA repair by RNA: templated, or not templated, that is the question. DNA Repair (Amst).

