# Supplementary material for: Choices have consequences: the nexus between DNA repair pathways and genomic instability in cancer
Source: Clin Transl Med. 2016 Dec 5;5:45. doi: 10.1186/s40169-016-0128-z (PMC5136664; doi:10.1186/s40169-016-0128-z)
Supplement: Supplementary file 1 — Additional file 1. Supplementary references. [file 40169_2016_128_MOESM1_ESM.doc]

**Supplementary Information**

**S1:**[http://www.businesswire.com/news/home/20160823006191/en/FDA-Accepts-Clovis-Oncology%E2%80%99s-Drug-Application-Rucaparib](http://www.businesswire.com/news/home/20160823006191/en/FDA-Accepts-Clovis-Oncology’s-Drug-Application-Rucaparib)

**S2:**<http://www.fda.gov/NewsEvents/Newsroom/PressAnnouncements/ucm427554.htm>

**S3:**<https://globenewswire.com/news-release/2016/06/29/852258/0/en/Myriad-s-myChoice-HRD-Test-Successfully-Identifies-Patients-that-Meet-Primary-Endpoint-in-TESARO-s-Pivotal-Phase-3-Ovarian-Cancer-Study-with-Niraparib.html>
